# The Role of Mitochondria from Mature Oocyte to Viable Blastocyst

**DOI:** 10.1155/2013/183024

**Published:** 2013-05-16

**Authors:** Scott Chappel

**Affiliations:** OvaScience, Inc., 215 First Street, Suite 240, Cambridge, MA 02142, USA

## Abstract

The oocyte requires a vast supply of energy after fertilization to support critical events such as spindle formation, chromatid separation, and cell division. Until blastocyst implantation, the developing zygote is dependent on the existing pool of mitochondria. That pool size within each cell decreases with each cell division. Mitochondria obtained from oocytes of women of advanced reproductive age harbor DNA deletions and nucleotide variations that impair function. The combination of lower number and increased frequency of mutations and deletions may result in inadequate mitochondrial activity necessary for continued embryo development and cause pregnancy failure. Previous reports suggested that mitochondrial activity within oocytes may be supplemented by donor cytoplasmic transfer at the time of intracytoplasmic sperm injection (ICSI). Those reports showed success; however, safety concerns arose due to the potential of two distinct populations of mitochondrial genomes in the offspring. Mitochondrial augmentation of oocytes is now reconsidered in light of our current understanding of mitochondrial function and the publication of a number of animal studies. With a better understanding of the role of this organelle in oocytes immediately after fertilization, blastocyst and offspring, mitochondrial augmentation may be reconsidered as a method to improve oocyte quality.

## 1. Introduction

Over the past decade, our understanding of mitochondrial function has matured. In addition to providing cellular energy in the form of ATP for almost all intracellular events, mitochondria have important functions in ion homeostasis, programmed cell death, and adaptive thermogenesis [1]. Mitochondrial dysfunction has been implicated in a number of pathophysiological processes such as aging, neurodegenerative diseases, diabetes and obesity, and infertility. This review will summarize the role of mitochondria in oocytes immediately prior to fertilization and up to the blastocyst stage. The concerns of cytoplasmic and mitochondrial transfer will be reconsidered in light of animal studies and our greater understanding of mitochondrial function to determine if it may be employed to improve fertility outcomes. 

## 2. Mitochondrial Structure and Function

Mitochondria are maternally inherited organelles that use high efficiency oxidative phosphorylation pathways to supply ATP for cellular energy demands. They are evolutionary relics of bacteria that invaded our ancestral cells about a billion years ago. These organelles exist in the cytoplasm of almost all eukaryotic cells and have a separate genome. The mitochondrial genome is a double stranded, circular DNA that is approximately 16.7 kb. Similar to prokaryotic DNA, human mitochondrial DNA (mtDNA) contains no introns. The mitochondrial genome replicates independently of the cell cycle. This DNA encodes enzymes involved in (aerobic) oxidative phosphorylation. This process provides a more efficient method for the production of ATP compared with the (anaerobic) glycolytic pathway.

The mitochondrial genome encodes 13 proteins (all part of the oxidative phosphorylation pathway), 22 transfer RNAs, and two ribosomal RNAs [2]. The expression of these gene products is controlled, in large part, by signals provided by the nucleus. Proteins encoded by nuclear DNA are imported into the mitochondria to control its function in a tissue-specific fashion [3, 4]. All of these nuclear-encoded proteins recognize specific mtDNA sequences and are thus dependent on optimal protein-protein as well as protein-DNA interactions [5]. As cellular demand increases, the nuclear genome produces mitochondrial regulatory factors that are imported into the mitochondria to initiate replication and transcription of mtDNA and expansion of the mitochondrial network.

Control of mitochondrial function is afforded not only by cell-specific mitochondrial transcription factors encoded in nuclear DNA [6] but also the availability of the precursor ADP and NADPH, substrates required for the synthesis of ATP. As NADPH levels decline, less ATP is produced [7]. In this way, mitochondrial function is regulated by substrate availability as well as highly specific communication between the mitochondrial and nuclear genomes. 

## 3. Mitochondrial Efficiency

The best-known function of mitochondria is the generation of ATP from food sources. Pyruvate, converted from glucose, is consumed by mitochondria to produce ATP. As mitochondria produce ATP, they release reactive oxygen species (ROS) locally that must be detoxified as they can induce oxidative damage to mitochondrial DNA (mtDNA). This damage results in mutations and deletions of mtDNA. The relative absence of repair enzymes for mtDNA may explain its sensitivity to oxidative stress-induced damage [8]. The 10- to 20-fold higher mutation rate in mitochondrial DNA compared with nuclear DNA is believed to be due to its proximity to ROS generation and the limited DNA repair capacity [9, 10]. As the organism, tissue, and cells age, exposure of the mitochondrial genome to ROS increases. This compromises the function of this organelle.

An accumulation of mutations in mtDNA may limit energy production. As a result, the cell has a decreased capacity to support all cellular events and especially normal chromosomal segregation during cell division. Many different mitochondrial deletions and mutations have been described. The most common is a 4,977 bp deletion that occurs within two 13 bp repeats (beginning at positions 8,470 and ending at 13,459 of the human mitochondrial genome) [11]. Accumulation of the 4,977 bp deletion within mtDNA represents a marker for aging [12–15]. 

## 4. Inheritance of Mitochondrial DNA

Unlike the nuclear genome that is transmitted to offspring through Mendelian inheritance patterns, most mammals inherit their mtDNA from the population that is present within the oocyte at the time of fertilization. The transmission of the maternal mitochondrial genome to the offspring is of great importance. During fertilization, mitochondria that are imported into the oocyte from the sperm are ubiquitinated and targeted for removal through the proteasome of the cell [16]. This assures that the source of mtDNA for the offspring will be solely maternal (homoplasmy). Multiple deletions of mitochondrial DNA have been reported in human sperm, especially in aging men or those with poor quality sperm [17]. It is conceivable that survival would be compromised if damaged paternal mitochondria were transmitted to the offspring. Negative physiological consequences of mitochondrial heteroplasmy in the offspring have been reported [18–20]. 

As the oocyte matures, the need for more energy requires a shift from glycolysis to oxidative phosphorylation. Energy needs peak at the time of ovulation [21]. Since embryonic mitochondrial replication does not occur until after the hatched blastocyst stage, mature (MII) oocytes, fertilized oocytes, and early cleavage stage embryos are dependent on the function of the mitochondrial pool present at ovulation [18]. Consequently, any adverse influence on mitochondrial function (i.e., accumulation of mutational load to the mtDNA) will negatively impact the development of the preimplantation embryo. Mitochondria with mutations in their genome present in the oocyte at the time of fertilization may be passed on to the offspring and be the basis for inheritance of debilitating or lethal metabolic diseases [22, 23]. 

## 5. Fertilization and the Importance of Mitochondria in Oocytes

The number of mitochondria within cells is often an indication of the activity of that cell. For instance, neurons, muscle cells, and mature oocytes have many copies of mtDNA compared with other somatic cells. ATP generating capability is critical for successful maturation of the cytoplasm and nucleus in preparation for fertilization and completion of meiosis II [13, 24, 25]. Good quality oocytes containing optimal mitochondrial numbers and sufficient levels of ATP (at least 2 pM) [26] produce higher quality blastocysts after fertilization [27].

Following fertilization and up to implantation, the embryo is dependent on the function of existing mitochondria. As cell division begins, the total amount of mitochondria within each blastomere decreases due to dilution with no new mitochondrial biosynthesis [24]. Early stage cells do not express the replication factors required to increase copy numbers of mitochondria. There is a dramatic reduction in the number of mitochondria per cell as the fertilized oocyte develops into a blastocyst. There are very few mitochondria per cell found in the hatched blastocyst (the bottle neck theory [28]) which is the mechanism for transmission of the highest quality, homoplasmic mtDNA to the offspring. At implantation, first the trophectoderm and then the entire embryo gain capacity to replicate mtDNA [24, 29].

In the early stage embryo, the mitochondria existing within the oocyte must provide adequate ATP to fuel the first few days of embryonic development [29, 30]. It is assumed that both anaerobic and aerobic respiration attempt to meet the energy needs of the early embryo. The embryo, however, cannot rely on anaerobic respiration when mitochondrial activity is insufficient due its low efficiency and end product inhibition. The role of mitochondrial oxidative phosphorylation and ATP production during the early fertilization and preimplantation process has been shown to be critical in many species [29, 31, 32] including humans [13]. Murine [33, 34], bovine [35], or porcine [32] oocytes treated with chemicals to impair mitochondrial function show dramatically compromised cleavage rates and increased incidence of aneuploidy. Oocytes are able to adjust local mitochondrial density within different intracellular regions to reflect the cell's changing intracellular needs [36]. The observed redistribution of mitochondria to spindles as well as microtubule organizing centers emphasizes this feature [36]. 

In addition to providing adequate energy at the appropriate intracellular location for cell division and chromosomal segregation in the oocyte, mitochondria also act to sequester calcium (cytoplasmic calcium buffer). Increases in cytoplasmic free calcium are essential for oocyte activation and embryo development after fertilization [22]. These calcium oscillations occur after sperm attachment to the oocyte membrane or after sperm injection at ICSI [37]. Fertilization completes meiosis II and requires increased amounts of ATP. The increase in calcium flux within the cell induces the expression of respiratory chain enzymes that increase ATP production through oxidative phosphorylation.

Mitochondria exhibit an interesting quality maintenance function. Similar to prokaryotes, mitochondria have numerous periods of fusion and fission. If active, mitochondria maintain a polarized membrane and fuse with other mitochondria, to transfer components and maintain or improve the function of damaged or poorly performing members [38]. If, however, the mitochondrion is not functional and its membrane is depolarized, it does not fuse with active ones and is targeted for removal. This process prevents the mixing of damaged with high-quality mitochondria and decreases the pool of poorly performing mitochondria [38].

## 6. Regulation of Mitochondrial Activity: Need for a Threshold 

Inadequate supplies of ATP will result in arrested development. There is a great interoocyte variability of ATP content within a cohort of oocytes when measured at one single time point. The cell regulates ATP synthesis tightly and a “snap-shot” of individual oocytes within a cohort may not accurately describe this dynamic process. Actual mitochondrial numbers are quite variables in all oocytes [13, 39, 40], suggesting that mitochondrial activity is not dictated solely by copy number. Adequate amounts of mitochondrial activity are required to provide the burst of activity that is, required up to the hatched blastocyst stage [41]. Mitochondrial activity is strictly regulated by nuclear signals, intracellular ion concentrations, and the availability of substrates [42, 43]. 

Sufficient amounts of ATP are required for numerous cellular events that are triggered after fertilization including polymerization of microtubules, cell cycle regulation, segregation of chromosomes, and membrane biosynthesis [44, 45]. Inappropriate mitochondrial activity at the pronuclear stage is associated with early developmental arrest and demise [46].

Since mitochondria contain multiple copies of DNA and cells may contain hundreds or thousands of mitochondria, a high proportion of mutations and deletions (mutational load) may be tolerated before a deficit in cellular function is apparent. Mitochondrial dysfunction is only revealed when overall mitochondrial function drops below a threshold [47]. When a follicle and oocyte are selected for final maturation and have inadequate mitochondrial activity, final maturation may be delayed or terminated. Delayed maturation may result in an increased rate of aneuploidy following fertilization and is directly related to a decline in pregnancy rate. Oocytic and embryonic aneuploidies are directly related to maternal age and mitochondrial activity [24, 44, 45].

## 7. Impact of Mitochondrial Insufficiency on Fertility

Aging is associated with a decrease in mitochondrial function, especially in nonreplicating cells such as the mature oocyte, and it is considered the basis of declining rates of fertility in women [7, 29, 45]. Age-related infertility is often due to poor oocyte quality rather than endometrial receptivity as demonstrated by the success of donor oocyte programs (http://apps.nccd.cdc.gov/art/Apps/NationalSummaryReport.aspx). 

Maternal age is associated with increased oxidative stress in oocytes and results in mitochondrial dysfunction. This dysfunction is due to oxidative damage, deletions, or point mutations and variations in the mitochondrial genome [12, 48, 49] and results in inadequate amounts of ATP as well as a deficit of other critical mitochondrial functions necessary postfertilization. Oocyte aging is due to general dysfunction of a number of cellular processes typical of cellular aging such as the ability to generate energy by mitochondria [13, 16, 39, 40, 50]. Thus, age-related reproductive failure is attributed primarily to the quality of the oocyte due at least in part to the accumulation of mtDNA damage [22, 50, 51]. Unlike the nuclear genome, the mitochondrial genome has a sub-optimal proofreading function, thus permitting a steady accumulation of mutations and deletions [8].

An example of mitochondrial insufficiency negatively affecting fertility is provided by studies of obesity. Infertility problems are common in obese women. Obesity induces altered mitochondrial function in cumulus cell-oocyte complex [52, 53]. The combination of increased lipid and fatty acid content and resultant increase in ROS in the ovary, oocyte, and surrounding cumulus cells negatively affects the function of these cells. This results in increased mitochondrial damage and decreased function. Oocytes that mature in this environment have reduced capacity for normal development [53].

Low ATP and decreased mtDNA copy number are associated with not only poor oocyte quality, but also a reduction in the quality of embryo development and suboptimal implantation and placentation rates [26, 41, 54]. With diminished numbers of mitochondria, the negative effects of poor mitochondrial quality (mutational load) may become more evident. The proportion of mutant/wild-type mitochondria in the oocyte may be low enough to allow for normal embryo development but be an explanation for the surprisingly high number of offspring diagnosed with mitochondrial disease [23]. 

To increase the probability of successful outcome in assisted reproduction, controlled ovarian hyperstimulation programs are routinely employed. These procedures themselves have been shown to decrease the quality of oocytes. Gonadotropin-induced hyperstimulation may further impair mitochondrial function within the oocyte. Monkey oocytes, collected after follicle stimulating hormone hyperstimulation, show a higher degree of the common deletion compared with age matched, unstimulated oocytes [55]. Similar results have been reported in the mouse [56, 57].

## 8. Oocyte Quality and Aneuploidy

Meiosis occurs two times during the maturation of oocytes, once at the time of ovulation (meiosis I) and the second at the time of fertilization (meiosis II). The first meiosis is ultimately completed when the mature oocyte is exposed to midcycle levels of LH. One of the most energy intensive activities within the oocyte is the assembly and disassembly of microtubules. This activity plays an important role in the proper positioning and segregation of chromosomes. The metaphase alignment of the chromosomes in the oocyte is dependent on the assembly and disassembly of microtubules [58]. As the oocyte ages, the ability to produce adequate spindle microtubules decreases and results in an increased incidence of aneuploidy or unequal development [59]. Meiotic division errors can lead to nonextrusion of the first polar body, irregular distribution of chromosomes, and aneuploidy. With advancing maternal age, the second meiosis becomes increasingly susceptible to aneuploidy compared to meiosis I [59–61]. Similar findings have been reported for the mouse [58]. 

Aneuploidy of the egg and embryo is the leading genetic cause of spontaneous abortions and developmental disabilities. The incidence of aneuploidy in eggs from women in their 20's is 2% but dramatically increases to 35% around 40 years of age and perhaps even higher [62]. The segregation and migration of chromosomes or chromatids to the appropriate daughter cell depend on binding of the DNA to spindles [63]. An inverse relationship exists between mitochondrial activity and the prevalence of chaotic mosaicism in the preimplantation embryo [64]. Competent mitochondrial activity includes migration to the appropriate site in the cell to provide local organelles with adequate amounts of energy. Thus, mitochondrial activity is a balance between functional capacity (mutational load), absolute mtDNA copy number within the cell, and motility of the organelle [22, 65–67].

Mitochondrial mutations and subsequent decline in ATP levels may accelerate follicular atresia and lead to premature ovarian failure. More specifically, lesions in the ATP synthase gene due to oxidative stress may be related to ovarian insufficiency and a loss of function [13, 50]. An increase in the 4977 bp deletion of mtDNA has been reported in ovarian tissue [68] as well as oocytes and embryos [55, 69, 70] and surrounding support cells of the follicle [71]. In addition, unfertilizable oocytes and developmentally arrested embryos show a decreased expression of mitochondrial genes and an increased presence of the 4977 bp deletion [72–74].

## 9. Third Party Cytoplasmic Transfer

In an effort to increase mitochondrial activity at the time of fertilization of the oocyte, ooplasm obtained from an oocyte donated by a younger woman was transferred to the oocyte of a reproductively mature woman. The technique of ooplasm transfer was based on a well-established method used in experimental embryology to induce the maturation of immature oocytes. This method was used with confidence as cytoplasmic manipulation of oocytes and early embryos was shown to be compatible with normal development [75]. 

Following donor cytoplasmic transfer during ICSI, improvements were observed in embryo formation, implantation, and live births [76–81]. Patient selection was not based solely upon maternal age but rather on those who had previously demonstrated poor embryo cleavage rates and morphological anomalies. These factors were considered to be representative of inadequate mitochondrial function. As live births of healthy children were achieved, this method was proposed to augment resident mitochondrial activity in compromised oocytes. 

## 10. Issues Related to Third Party Cytoplasmic Transfer

While the benefit of cytoplasmic transfer in fertility enhancement was observed, issues related to the “3 parent genome” arose. As detailed previously, significant effort is expended by the fertilized oocyte to maintain the maternal identity of its mitochondria. There is a great deal of communication between the nuclear and mitochondrial genomes and strict control over mitochondrial function through this communication. Infusion of third party mitochondria itself or with deletions and/or mutations could theoretically increase the risk for heritable mitochondrial disease [82]. It has been shown that some children born using the transfer of third party cytoplasm exhibited mitochondrial heteroplasmy [83]. Since the effect of two different mitochondrial genomes on the health of the offspring was unknown, the US Food and Drug Administration reviewed the technique in 2002. They suggested that the technique of donor cytoplasm transfer be suspended pending the successful completion of studies under an approved Investigational New Drug application. 

Although cytoplasmic transfer using donor material has been shown to improve the success of IVF, a study in mice demonstrated a range of physiological abnormalities in offspring when equal proportions of heterogeneous populations of mitochondria were generated in the oocyte [19]. Metabolic abnormalities in offspring may have been due to the differences in response to nuclear signals between the two populations of mitochondrial DNA. Due to intimate communication with the nucleus, mitochondria optimally should be obtained from the same female providing the oocyte [82]. Similar metabolic effects were observed when offspring of mice carrying a significant load of mutated mitochondria were examined [84]. These murine studies demonstrated that the addition of supplemental mitochondria can negatively affect outcomes of offspring unless they are obtained from the same genetic source and are mutation-free.

## 11. Other Methods to Increase Intracellular Mitochondrial Activity in Oocytes

Dietary supplementation with CoQ10 may increase mitochondrial activity within the oocyte and developing embryo [85]. CoQ10 is a coenzyme that aids in the transport of electrons along the mitochondrial respiratory chain. Since tissue levels of CoQ10 decrease with age, supplementation of this agent may improve mitochondrial function in the ovary. CoQ10 administration is effective in the treatment of a variety of pathological conditions, including hypertriglyceridemia and Friedrich's ataxia. Dietary supplementation with CoQ10 may improve oocyte and embryo quality, especially in women of advanced reproductive age.

## 12. The Safety and Efficacy of Cytoplasmic or Mitochondrial Transfer: Animal Studies

Since these initial studies in humans, a large compendium of reports in animals have shown that the transfer of cytoplasm or an enriched fraction of mitochondria can reduce in vitro fragmentation of oocytes and increase cleavage rates of recipient embryos compared with noninjected controls. These studies also have demonstrated that this technique can produce healthy embryo and offspring (Table 1). 

*Mouse*. Oocytes that have had their mitochondria damaged or have not converted from glycolytic to aerobic respiration have a lower likelihood of development after fertilization [29, 86]. Early studies demonstrated the feasibility of injection of mitochondria into an oocyte to increase intracellular ATP concentrations [87]. Mitochondrial injections increase the viability of mouse oocytes destined for apoptotic cell death [88]. When mitochondria were stained with green fluorescent protein (GFP) prior to transfer to the oocyte, GFP-marked mitochondria were observed until blastocyst stage [89]. Following infusion of GFP mitochondria, dense clustering of mitochondria occurred around spindles, where ATP was needed. 

Injection of mitochondria into mouse oocytes did not negatively affect survival rate or development rate to morula stage compared with buffer-injected controls [90]. No long-term effects on growth of offspring or phenotypic abnormalities were observed when intra- and interstrain cytoplasmic transfers were compared [91]. When mitochondrial concentrates were injected into mouse 2 pronuclear stage embryos, there was an increase in progression to blastocyst stage with no untoward effects [92]. These authors commented on the need to avoid heteroplasmy of mitochondria to maintain appropriate nuclear-mitochondrial communication required for optimal embryo development.

*Rabbit*. Transfer of homoplasmic cytoplasm into a high quality MII oocyte did not affect survival of oocyte, fertilization rate or progression to 2 or 8 cell stage, and morula or blastocyst stage of embryo [93]. On the other hand, transfer of heteroplasmic ooplasm resulted in a decrease in the number of fertilized oocytes reaching the blastocyst stage. The authors conclude that transfer of homogeneous cytoplasm is required for optimal preimplantation embryo development [93].

*Cow.* As with other species, oocyte and embryo quality as well as reproductive outcome depend in part on the quality of mitochondria and ATP content in the oocyte [94, 95]. Treatment of bovine oocytes with ethidium bromide depletes mtDNA content in oocytes, and as a result, they are arrested in pre-implantation development [20, 35]. Cytoplasmic transfer to these impaired oocytes resulted in the complete rescue of the oocytes, which then are developed into normal calves.

Treatment of poor quality bovine oocytes with mitochondria obtained from the animal's own granulosa cells resulted in dramatic improvements in oocyte quality as well as rates of morula, blastocyst, and hatched blastocysts [100]. Addition of mitochondria obtained from the same breed improved embryo quality during preimplantation development. There is a segregation of donor mitochondria by the oocyte after cytoplasmic transfer [20] supporting the hypothesis that mitochondrial homoplasmy is optimal.

*Pig*. Supplementation of developmentally incompetent oocytes by injection of mitochondria resulted in a doubling of mitochondria number and normal embryo development. As a result fertilization rates were doubled or tripled (from approximately 10–20% to 30–40%) [31, 32].

## 13. Summary of Animal and Human Studies

Supplementation of mitochondrial activity in oocytes by transfer of cytoplasm or mitochondria improves fertility in all species tested including humans. Following mitochondrial transfer, these organelles are detectable at least until the blastocyst state and may be found in the offspring. Suboptimal results were achieved when the mitochondria used were obtained from somatic sources, were genetically different from resident mitochondria, or contained deletions or mutations. These studies suggest that while the transfer of mitochondria to oocytes can improve oocyte quality and reproductive success, the source and quality of mitochondria may affect the safety of the offspring.

## 14. Why Not Mitochondria from the Patient's Own Somatic Cells? 

In general, most somatic cells that would be obtained from the reproductively mature oocyte donor would be expected to contain mitochondrial DNA deletions and mutations [14, 15]. Such mitochondrial DNA would not be considered safe for the offspring, as the transmission of these mutations and deletions to all embryonic tissue would be a potential risk [23]. Somatic cell mitochondria appeared to adversely affect embryonic development [24, 90, 97]. 

Autologous mitochondria obtained from the patient's own granulosa cells have been used to successfully improve fertility outcomes [101]. However, concerns were raised about the use of somatic cell mitochondria, as they would be expected to contain point mutations and deletions, similar to all other somatic cells [24, 71, 103]. In addition, death (apoptotic) signals from mitochondria within granulosa cells control follicular atresia and prevent oocyte development [104, 105]. Thus, their transfer during ICSI could prove to be detrimental to the oocyte.

## 15. Are Tissue-Specific Mitochondria Required?

While over 1,500 proteins are present within the mitochondria, only 13 are encoded within the mitochondrial genome [24, 106, 107]. Thus, most mitochondrial proteins are imported from the cytoplasm and regulate mitochondrial function in a tissue-specific fashion [3, 4, 106]. The mitochondrial proteome is cell specific and reflects the needs of the cell in which it resides. 

Mitochondrial function varies widely between cells and tissue types [1, 108]. As a result, the cell type used as a source of mitochondria for oocyte transfer should be closely related to the recipient cell, so that the coordination of mtDNA and nuclear DNA communication has already occurred. Thus, mitochondria used for oocyte augmentation should be obtained from ovarian, or more preferably, oocyte-differentiated cells. These facts make it imperative that mitochondria used to improve bioenergetics of the oocyte be sourced from cells of oogonial derivation.

## 16. Use of Germ Line Quality Mitochondria: Oocyte Precursor Cells

High-quality, autologous mitochondria must be used for energy augmentation of oocytes through mitochondrial transfer. The transfer of the same mtDNA obtained from oocyte precursor cells would reduce possible compromised oxidative phosphorylation function that could arise through mixing mtDNA genotypes [19, 31]. The existence of oocyte precursor cells has been reported in both the mouse and human [109–115]. While the previous independent observations argue strongly for the existence of these cells in the mouse or human ovarian epithelium, there have been reports to the contrary [116]. There is also a debate in the literature as to the role of these cells in normal folliculogenesis [117]. Due to the fact that these cells have differentiated to the oocyte lineage but have not spent years in a postmitotic state, these cells may be used as a source of tissue-specific, autologous mitochondrial DNA free of mutations and deletions. 

Mechanisms exist within the oocyte to amplify high-quality mitochondria DNA even in the presence of mutated variants [28]. Provision of germ line quality mitochondria, through the use of the patient's own cells, can be expected to provide necessary ATP as well as increase the proportion of high-quality, homoplasmic mtDNA in the oocyte [84]. The frequency of mitochondrial disease increases in children conceived from women of advanced reproductive age [118, 119]. In an effort to improve the bioenergetics of the oocyte by mitochondrial supplementation, it is critical that the autologous and tissue-specific mitochondria be of the highest quality: free of DNA mutations and deletions. 

## 17. Severe Mitochondrial Mutations and Deletions

Numerous mitochondrial mutations, deletions, and nucleotide variations are found in oocytes and blastocysts [49], especially in women of advanced reproductive age, some of which may be considered unsafe for transmission to offspring. New therapies are being developed to allow carriers of mitochondrial disease to have their own genetic child. In this case, the nucleus of the patient's oocyte may be transferred into an enucleated donor oocyte with normal mitochondria. Studies have shown that a low amount (<1%) of mitochondrial DNA is transferred with the nucleus [49, 120], and differentiated cells derived from these blastocysts exhibited no evidence of transmission of the patient's mitochondrial DNA. These studies demonstrate the promise of nuclear genome transfer to prevent mitochondrial disease transmission in affected patients.

## 18. Concluding Remarks

Augmentation of the energy in the oocyte by cytoplasmic or mitochondrial injections has been shown to improve fertility outcomes. Human and animal studies have established the rules by which this procedure can be safely performed.

- The mitochondria must be obtained from the patients own cells, such that communication between the mtDNA and nuclear DNA is optimal (maintenance of homoplasmy in the offspring)
- The cell source of mitochondria should optimally be of ovarian and more preferably, oocyte origin such that the mitochondria are prepared to functionally respond to tissue- and cell-specific demands.
- The mitochondrial genome of these cells must be of high quality, without deletions or mutations so that no mutations are transmitted to the offspring.

Over the past few years, several laboratories have reported the identification and characterization of oocyte precursor cells and developed methods to isolate these cells from ovarian tissue. These germ line cells can serve as a source of patient- and tissue-specific mitochondria that may be used for oocyte augmentation. Mitochondria can be isolated and delivered at the time of ICSI to the oocyte with inadequate levels of mitochondrial activity. This method satisfies the rules established in preclinical and clinical studies and is proposed to be a safe and effective means to improve oocyte and preimplantation embryo quality in an assisted reproductive technology setting. 

## Figures and Tables

**Table 1:** Summary of Previous Cytoplasm and Mitochondrial Transfer Studies.

Species	Cytoplasm (Cyto) or mitochondria (Mito) transfer	Safe for oocyte	Increased fertilization rate	Viable blastocyst	Reference
Mouse	Cyto and Mito	*✓*	*✓*	*✓*	[87, 89–92, 96–99]
Pig	Cyto	*✓*	*✓*	*✓*	[31]
Cow	Cyto and Mito	*✓*	*✓*	*✓*	[20, 35, 100]
Human	Cyto and Mito	*✓*	*✓*	*✓*	[78–81, 101, 102]
